# Skeletal Muscle-Specific Expression of PGC-1α-b, an Exercise-Responsive Isoform, Increases Exercise Capacity and Peak Oxygen Uptake

**DOI:** 10.1371/journal.pone.0028290

**Published:** 2011-12-08

**Authors:** Miki Tadaishi, Shinji Miura, Yuko Kai, Yutaka Kano, Yuichi Oishi, Osamu Ezaki

**Affiliations:** 1 Department of Nutritional Science, National Institute of Health and Nutrition, Tokyo, Japan; 2 Department of Nutritional Science, Faculty of Applied Bioscience, Tokyo University of Agriculture, Tokyo, Japan; 3 Department of Engineering Science, University of Electro-Communications, Tokyo, Japan; Universidad Europea de Madrid, Spain

## Abstract

**Background:**

Maximal oxygen uptake (VO_2max_) predicts mortality and is associated with endurance performance. Trained subjects have a high VO_2max_ due to a high cardiac output and high metabolic capacity of skeletal muscles. Peroxisome proliferator-activated receptor γ coactivator 1α (PGC-1α), a nuclear receptor coactivator, promotes mitochondrial biogenesis, a fiber-type switch to oxidative fibers, and angiogenesis in skeletal muscle. Because exercise training increases PGC-1α in skeletal muscle, PGC-1α-mediated changes may contribute to the improvement of exercise capacity and VO_2max_. There are three isoforms of PGC-1α mRNA. PGC-1α-b protein, whose amino terminus is different from PGC-1α-a protein, is a predominant PGC-1α isoform in response to exercise. We investigated whether alterations of skeletal muscle metabolism by overexpression of PGC-1α-b in skeletal muscle, but not heart, would increase VO_2max_ and exercise capacity.

**Methodology/Principal Findings:**

Transgenic mice showed overexpression of PGC-1α-b protein in skeletal muscle but not in heart. Overexpression of PGC-1α-b promoted mitochondrial biogenesis 4-fold, increased the expression of fatty acid transporters, enhanced angiogenesis in skeletal muscle 1.4 to 2.7-fold, and promoted exercise capacity (expressed by maximum speed) by 35% and peak oxygen uptake by 20%. Across a broad range of either the absolute exercise intensity, or the same relative exercise intensities, lipid oxidation was always higher in the transgenic mice than wild-type littermates, suggesting that lipid is the predominant fuel source for exercise in the transgenic mice. However, muscle glycogen usage during exercise was absent in the transgenic mice.

**Conclusions/Significance:**

Increased mitochondrial biogenesis, capillaries, and fatty acid transporters in skeletal muscles may contribute to improved exercise capacity via an increase in fatty acid utilization. Increases in PGC-1α-b protein or function might be a useful strategy for sedentary subjects to perform exercise efficiently, which would lead to prevention of life-style related diseases and increased lifespan.

## Introduction

Cardiorespiratory fitness (CRF) is a strong and independent predictor of all-cause and cardiovascular disease mortality [1], [2]. A recent meta-analysis showed that compared to participants with high CRF, those with low CRF had a relative risk for all-cause mortality of 1.70 (95% CI, 1.51–1.92; *P*<0.001) and for coronary heart disease (CHD)/cardiovascular disease (CVD) events of 1.56 (95% CI, 1.39–1.75; *P*<0.001) [3]. Maximal O_2_ uptake (VO_2max_), the maximal amount of O_2_ per unit of time, is considered the gold standard measure of CRF. Central O_2_ delivery from the heart and peripheral use of O_2_ in skeletal muscle contribute to training-induced changes in VO_2max_, although the genetic contribution of VO_2max_ was estimated at 47% [4], [5]. The pump capacity of the heart is considered to be a primary determinant of VO_2max_ in humans [6]. On the other hand, in subjects receiving a different intensity of exercise, muscle citrate synthase (CS) activity, a determinant of mitochondrial number (or volume), was closely correlated to VO_2max_ [7], [8]. More recently, aerobic interval training was shown to improve not only maximal stroke volume (measured as peak O_2_ pulse) but also Ca^2+^ cycling and mitochondrial capacity in skeletal muscle (assessed by improved sarcoplasmic reticulum ATPase capacity and peroxisome proliferator-activated receptor γ coactivator 1α (PGC-1α) levels in skeletal muscle) to a larger extent than continuous moderate exercise in heart failure and metabolic syndrome patients [9], [10]. However, it is not clear whether increased mitochondrial capacity, fatty acid oxidation and blood flow in skeletal muscle in response to exercise training contribute to an increase in VO_2max_.

PGC-1α is induced by exercise and promotes mitochondrial biogenesis, a fiber-type switch to oxidative fibers, and angiogenesis in skeletal muscle [11], [12]. There are three isoforms of PGC-1α mRNA. PGC-1α-a is transcribed from exon 1a. PGC-1α-b and PGC-1α-c are transcribed from an alternate exon, 1b. PGC-1α-a encodes a protein of 795 amino acids [12]–[14]. All of these promote mitochondrial biogenesis and angiogenesis in skeletal muscles. However, sixteen amino acids at the amino terminus in PGC-1α-a differ from PGC-1α-b and PGC-1α-c, and this small difference may affect the binding to putative transcription factors, possibly leading to different metabolic responses. We previously reported that exercise-induced expression of isoforms of PGC-1α mRNA was dependent on the intensity of exercise [15]. A single bout of low-intensity exercise could increase PGC-1α-b and PGC-1α-c mRNAs via β2-adrenergic receptor (AR) activation, whereas an increase in PGC-1α-a mRNA expression required high-intensity exercise and was independent of β2-AR activation. Among the PGC-1α isoforms, the increase in PGC-1α-b expression was the largest in response to bouts of exercise [15].

Calvo et al. addressed the effects of increased muscle PGC-1α-a levels on exercise performance [16]. Mice with muscle-specific overexpression of PGC-1α-a (MCK-PGC-1α-a) demonstrated an improvement in exercise performance and also exhibited ∼20% higher peak oxygen uptake than wild-type mice. However, the MCK-PGC-1α-a mice did have elevated PGC-1α-a mRNA levels in the heart, and it may thus be speculated that this was sufficient to elicit the observed effects on peak oxygen uptake.

The generation of transgenic mice overexpressing PGC-1α-b in skeletal muscle but not in the heart may help to elucidate the role of PGC-1α in the improvement of exercise performance and increases in VO_2_max. To clarify this, we analyzed transgenic mice that overexpressed PGC-1α-b, the most exercise-inducible isoform, in skeletal muscle.

## Methods

### Transgenic mice

The methods for the generation of transgenic mice overexpressing PGC-1α-b in skeletal muscle were described previously [13]. The human α-skeletal actin promoter provided by Drs. E. C. Hardeman and K. L. Guven (Children's Medical Research Institute, Australia) was used to express PGC-1α-b in skeletal muscle. The transgenic mice (heterozygotes, BDF 1 background) and wild-type C57BL6 mice were crossed and the offspring (heterozygote and wild-type, born at the same period) were used for the experiments. Mice were cared for in accordance with the NIH Guide for the Care and Use of Laboratory Animals and our institutional guidelines. All animal experiments were conducted with the approval of the National Institute of Health and Nutrition Ethics Committee on Animal Research (approval ID: No 908, 1008, and 1111).

### Body Composition Analysis

Mice were scanned with a Lunar PIXI mus2 densitometer (Lunar Corporation, Madison, WI), equipped for dual energy X-ray absorptiometry (DEXA) [17].

### Quantitative Real-time RT-PCR

RNA preparation methods and quantitative real-time RT-PCR were performed as described previously [13]. The mouse-specific primer pairs used were as shown in Table 1.

**Table 1:** Mouse-specific primer pairs used for quantitative RT-PCR.

	Forward	Reverse
36B4	5′-GGCCCTGCACTCTCGCTTTC-3′	5′-TGCCAGGACGCGCTTGT-3′
PGC-1α	5′-CGGAAATCATATCCAACCAG-3′	5′-TGAGGACCGCTAGCAAGTTTG-3′
COX2	5′-CCGACTAAATCAAGCAACAGTAACA-3′	5′-AAATTTCAGAGCATTGGCCATAG-3′
COX4	5′-CTATGTGTATGGCCCCATCC-3′	5′-AGCGGGCTCTCACTTCTTC-3′
MHC1	5′-CCAAGGGCCTGAATGAGGAG-3′	5′-GCAAAGGCTCCAGGTCTGAG-3′
MHC2A	5′-AAGCGAAGAGTAAGGCTGTC-3′	5′-GTGATTGCTTGCAAAGGAAC-3′
MHC2X	5′-AGGCCAGGGTCCGTGAA-3′	5′-CCACGTTGCGCTTCTGTTC-3′
MHC2B	5′-ACAAGCTGCGGGTGAAGAGC-3′	5′-CAGGACAGTGACAAAGAACG-3′
GYS1	5′-CCCCCAACTCCGGAACTG-3′	5′-AATACACCTTACAACCCTTGCTGTT-3′
PHKA1	5′-AGCGTTCGTCCCACTGATTC-3′	5′-GCTCCAACAGCACCAATCTCA-3′
PYGM	5′-GCATCAAAAGGGAGCCCAAT-3′	5′-GCCTTGCCTCCAATCATGA-3′
GLUT4	5′-ATGGCTGTCGCTGGTTTCTC-3′	5′-ACCCATGCCGACAATGAAGT-3′
HK2	5′-GGAACCCAGCTGTTTGACCA-3′	5′-CAGGGGAACGAGAAGGTGAAA-3′
PFKM	5′-GCCATCGCCGTGTTGAC-3′	5′-GCCCTGACGGCAGCATT-3′
PFKFB3	5′-ACGAAGATGCCGTTGGAACT-3′	5′-TCGACGGGCACCCAGAT-3′
PKM2	5′-TTGACTCTGCCCCCATCAC-3′	5′-GCAGGCCCAATGGTACAAAT-3′
PDK4	5′-AGCTGGTGAAGAGCTGGTATATCC-3′	5′-TCTGGTCTTCTGGGCTCTTCTC-3′
LPL	5′-CCATGGATGGACGGTAACG-3′	5′-TACAGGGCGGCCACAAGT-3′
CD36	5′-CGGAACTGTGGGCTCATTG-3′	5′-GCATGAGAATGCCTCCAAACA-3′
FATP1	5′-TGACAGTGCCACCAACAAGAA-3′	5′-GCGCTATCGCCCTTTCG-3′
FABP-pm	5′-CGAGCAGGGCATCAATGTCT-3′	5′-CCGTACAGGCCCATGTTCTT-3′
FABP3	5′-ACTCGGTGTGGGCTTTGC-3′	5′-ATGATGGTAGTAGGCTTGGTCATG-3′
CPT1	5′-GTGCAAGCAGCCCGTCTAG-3′	5′-TTGCGGCGATACATGATCAT-3′
MCAD	5′-TGGATCTGTGCAGCGGATT-3′	5′-GGGTCACCATAGAGCTGAAGACA-3′
Mb	5′-GGCAGCTGGTGCTGAATGT-3′	5′-TAAACAGACCGATGAGGACTTCCT-3′
VEGF-A	5′-CGAGATAGAGTACATCTTCAAGCC-3′	5′-TCATCGTTACAGCAGCCTGC-3′
VEGF-B	5′-AAAAAAAAAGGAGAGTGCTGTGAAG-3′	5′-TCCCAGCCCGGAACAGA-3′
PDGF-B	5′-GGTCAGCGCCGAGGG-3′	5′-GCGGATGGAGTGGTCGC-3′
nNOS	5′-AAGGAGCAAGGAGGCCATAT-3′	5′-ATATGTTCTGAGGGTGACCCC-3′
eNOS	5′-AAGGAAGTAGCCAATGCAGTGAA-3′	5′-TCACACGCTTCGCCATCA-3′
Nur77	5′-GGGTGACCCCACTATTTGTC-3′	5′-CGGAAGAGATCTCGAGTTGG-3′

PGC, peroxisome proliferator-activated receptor γ coactivator; COX, cytochrome c oxidase; MHC, myosin heavy chain; GYS, glycogen synthase; PHKA, phosphorylase kinase alpha, PYGM, muscle glycogen phosphorylase; GLUT, glucose transporter; HK, hexokinase; PFKM, muscle phosphofructokinase; PFKFB, 6-phosphofructo-2-kinase/fructose-2,6-biphosphatase; PKM, muscle pyruvate kinase; PDK, pyruvate dehydrogenase kinase; LPL, lipoprotein lipase; FATP, fatty acid transport protein; FABP-pm, plasma membrane fatty acid binding protein; FABP, fatty acid binding protein; CPT, carnitine palmitoyltransferase; MCAD, medium chain acyl-CoA dehydrogenase; Mb, myoglobin; VEGF, vascular endothelial growth factor; PDGF, platelet-derived growth factor; nNOS, neuronal nitric oxide synthase; eNOS, endothelial nitric oxide synthase.

### Western blot

Frozen skeletal muscle and heart was powdered under liquid nitrogen, and then homogenized in RIPA Lysis Buffer (Upstate Bitechnology, Lake Placid, NY) containing 1 mM sodium orthovanadate, 1 mM sodium fluoride, and the “Complete Mini, EDTA-free” protease inhibitor cocktail (1 tablet per 10 ml). After three cycles of freezing and thawing, the supernatant was separated by centrifugation at 20,400 g for 15 min at 4°C. Fifteen µg of protein from the supernatant was applied onto an SDS-PAGE gel. PGC-1α protein was identified by Western blotting with anti-PGC-1α (Rabbit polyclonal IgG) against the carboxyl terminus, 777–797 (Calbiochem, San Diego, CA).

### Measurement of mitochondrial DNA copy number and CS activity

The mitochondrial DNA copy number was normalized to the copy number of a gene contained in the nuclear genome. The mitochondrial DNA copy number in PGC-1α-b transgenic mice was expressed as a percentage of the copy number in control wild-type mice. Real-time PCR was used to estimate the copy number of specific genes in skeletal muscles as described previously [18]. CS activity was measured as described previously [19].

### Preparation of the mitochondrial fraction from skeletal muscle

To obtain a pure and intact mitochondrial fraction, differential centrifugation was used [20]–[22]. All procedures were performed at 0–4°C. Media used were as follows: medium I: 100 mM KCl, 5 mM MgSO_4_·7H_2_O, 5 mM EDTA, and 50 mM Tris·HCl, pH 7.4; medium II: medium I plus 1 mM ATP, pH 7.4; medium III: 220 mM sucrose, 70 mM mannitol, 10 mM Tris·HCl, and 1 mM EDTA, pH 7.4. Fresh skeletal muscle was immediately placed in ice-cold medium I and then blotted and weighed. Muscle was minced with scissors in 1 ml of medium II. Minced muscle was homogenized in 20 vol of ice-cold medium II using a Teflon pestle. The homogenate was treated with a protease (Sigma P5380, 0.025 ml/g tissue) for 5 min to digest the myofibrils. Addition of 10 ml of ice-cold medium II was used to diminish the action of the protease. Samples were spun at 5,000 g for 5 min, and the supernatant was removed. The pellet was resuspended in 10 vol of medium II and spun at 800 g for 10 min. The mitochondria found in the supernatant were spun at 12,000 g for 10 min. The pellet was washed in medium II and spun at 12,000 g for 10 min at 4°C. The pellet was resuspended in 1 µl medium III/mg tissue.

### Measurement of mitochondrial respiration

Mitochondrial oxygen consumption was measured polarographically using an Oxygraph oxygen electrode unit (Hansatech Instruments, Kings Lynn, UK) at 30°C. The respiration was measured in a medium containing 225 mM mannitol, 75 mM sucrose, 10 mM KCl, 10 mM Tris-HCl, and 5 mM KH_2_PO_4_, pH 7.2 [23]. For the measurement of pyruvate and malate oxidation, the following substrate combinations were used: 5 mM pyruvate+5 mM malate+1 mM ATP. Oxidative phosphorylation (state III respiration) was initiated by adding 125 nmol ADP in the presence or absence of 5 µg/ml oligomycin.

### Histological Analyses

Samples of the tibialis anterior (TA) muscle at 10 weeks of age were frozen in liquid nitrogen-cooled isopentane. The identification of capillaries was performed using the anti-CD31 antibody (#553370, BD-Pharmingen, Franklin Lakes, NJ, USA), which recognizes platelet endothelial cell adhesion molecule-1(PECAM), a transmembranous glycoprotein strongly expressed by vascular endothelial cells [24], [25]. The acetone fixed slides were incubated for 1 h with anti-CD31 antibody and for 30 min with Simple Stain Mouse MAX PO (rat) (Nichirei, Tokyo, Japan). Visualization of antibody staining was achieved using 3,3′-diaminobenzidine tetrahydrochloride as the chromogen. Capillary density (number/mm^2^) and the capillary-to-fiber ratio were measured directly from images of micrographs projected on a screen.

### Measurement of spontaneous physical activity

To examine spontaneous physical activity, mice were housed in cages equipped with an infrared sensor, the activity was measured for 24 h, and the data were analyzed using a Supermex system (Muromachi Kikai, Tokyo, Japan).

### Measurement of exercise capacity

Exercise capacity was determined by an exercise tolerance test during the peak oxygen uptake challenge as described previously [16] with modifications (no incline in our protocols). To determine peak VO_2_, mice were placed in an air-tight treadmill (Muromachi Kikai) for 60 min at 0 m/min. The mice were then challenged at 10 m/min. The speed increased 2 m/min every 3 min until exhaustion. The mice ran until exhaustion, which is defined as remaining on the shocker plate for more than 10–15 s, as described previously [16]. Please make a note that the definition of exhaustion was not the same as used in other studies, in which volitional fatigue was taking into consideration [26], [27].

### Measurement of oxygen consumption and carbon dioxide production

Open-circuit indirect calorimetry was performed with an O_2_/CO_2_ metabolism measuring system for small animals (MK-5000RQ; Muromachi Kikai). The system monitored VO_2_ and VCO_2_ at 3-min intervals and calculated the respiratory quotient (RQ) ratio (VCO_2_/VO_2_). Measurements were performed during the dark (from 19:00 to 7:00) or light (from 7:00 to 16:30) period under fasting conditions.

For measurement of VO_2_ and VCO_2_ during exercise, mice were allowed to acclimatize to the air-tight treadmill chamber (Muromachi Kikai) for 30 min, at which point VO_2_ and VCO_2_ were stable. Measurements were continued for another 30 min while mice were maintained in a sedentary state. Mice were then exercised as described above.

The substrate utilization rate and energy production rate were calculated using the formula used by Ferrannini where the rate of glucose oxidation (g/min) = 4.55VCO_2_ (l/min)-3.21VO_2_ (l/min)-2.87N(mg/min), the rate of lipid oxidation (g/min) = 1.67 (VO_2_-VCO_2_)-1.92N, and the rate of energy production (kcal/min) = 3.91 VO_2_+1.10VCO_2_-3.34N, and where N is the rate of urinary nitrogen excretion used to estimate protein oxidation [28]. However, considering that only a small portion of resting and exercise energy expenditure arises from protein oxidation, the contributions of protein oxidation were ignored [29].

### Other assays

Glycogen content in the gastrocnemius and liver was measured as glycosyl units after acid hydrolysis [30]. Blood glucose concentration was measured with a glucose analyzer (Glucometer DEX; Bayer Medical, Tokyo, Japan). Lactate levels were measured by Lactate Pro (Arkray, Kyoto, Japan). Blood samples were obtained by cutting the tail tip.

### Statistical analysis

Data were analyzed by one-way ANOVA. Where differences were significant, each group was compared with the other by Student's *t* test (JMP 5.1.2; SAS, Campus Drive, Cary, NC, USA). In the exercise tolerance test, a Kaplan-Meier survival curve was obtained, and a comparison of groups was performed using the log-rank test (StatView 5.0). Statistical significance was defined as *P*<0.05. Values are shown as mean ± SE.

## Results

### Skeletal muscle-specific expression of PGC-1α-b increases the biogenesis of mitochondria in skeletal muscles but not in heart

**Figure 1:**
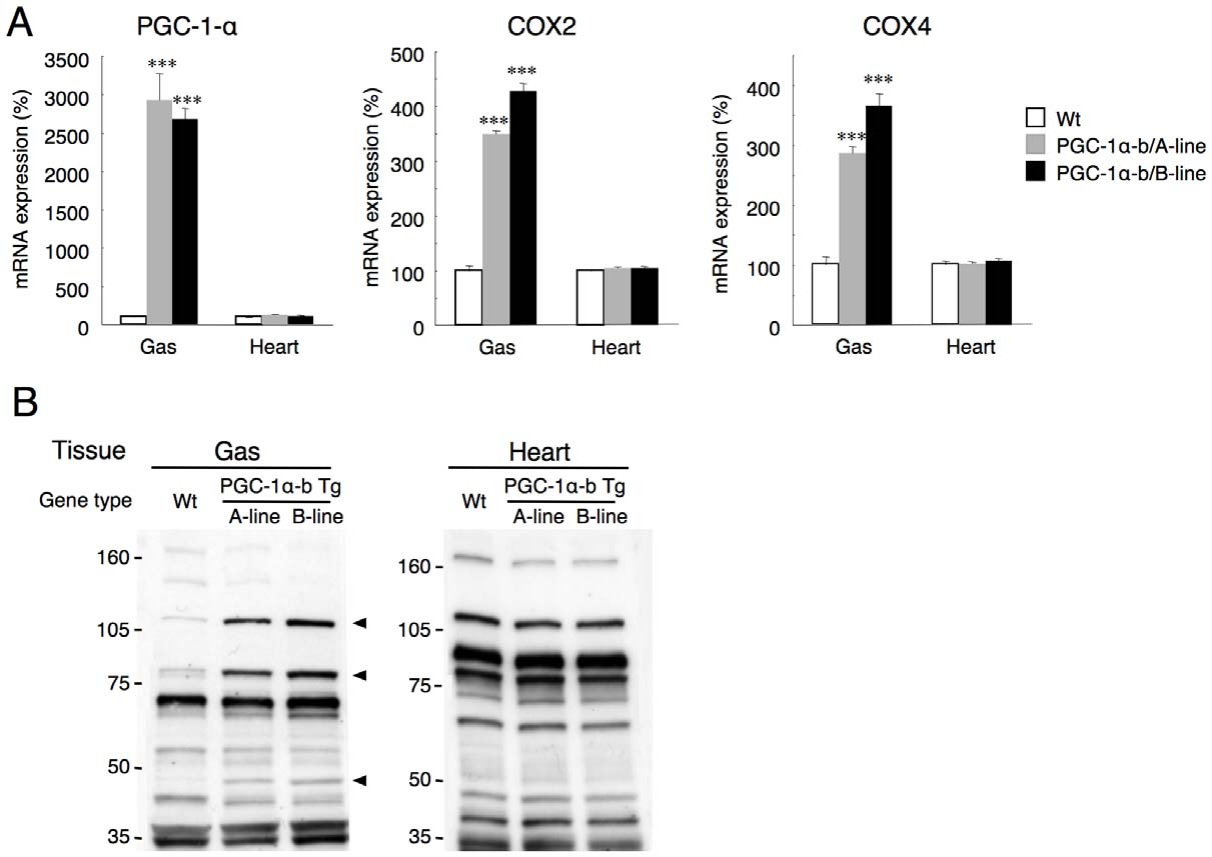
Skeletal muscle-specific expression of PGC-1α-b. (A) Total expression of PGC-1α and its target genes, COX2 and COX4, in wild-type (Wt) and PGC-1α-b mice (A line and B line) at 25 weeks of age (n = 3) was measured by quantitative real-time RT-PCR in skeletal muscle (gastrocnemius, Gas) and heart. Values are means ± SE. ***P<0.001 vs. Wt. (B) Total lysates from skeletal muscle (Gas) and heart were subjected to SDS-PAGE followed by Western blot analysis with anti-PGC-1α antibodies (n = 3). Typical blots are shown. For skeletal muscle samples, densitometric analysis was performed on the bands with increased intensity (arrow heads) following skeletal muscle-specific overexpression of PGC-1α-b.

Skeletal muscle specific PGC-1α-b mice were made with a DNA construct containing the 5′-flanking skeletal muscle-specific regulatory region and the promoter of the human α-skeletal actin gene, and a cDNA encoding a PGC-1α-b [13]. Quantitative real-time RT-PCR showed that PGC-1α mRNA was expressed 29.2- and 26.8-fold higher in skeletal muscles of transgenic lines A and B, respectively, than in wild-type mice, but there was no difference in heart muscle (Fig. 1A).

PGC-1α protein was identified by Western blot analysis with an antibody against the carboxyl terminus of the PGC-1α-a protein, because the carboxyl terminus is the same in all PGC-1α isoforms (the amino terminus of PGC-1α-b protein differs from PGC-1α-a protein) [13]. In previous studies on mouse skeletal muscle and cultured cells, this antibody detected a 113 kDa protein that was considered the full length PGC-1α-a protein [31], [32]. In skeletal muscle taken from the transgenic mice in this study, increased labeling of the bands at 110 kDa (16.6-fold in line A and 24.8-fold in line B), 85 kDa (4.5-fold in line A and 5.5-fold in line B) and 45 kDa (5.8-fold in line A and 10.9-fold in line B) were detected with this antibody (Fig. 1B). In the transgenic mice, a decrease in the 40 kDa band was also observed. This might be due to the effects of alternate splicing of endogenous PGC-1α, as suggested in a previous study [32]. In heart, no significant change was observed between the genotypes, which confirmed that PGC-1α-b protein was not over-expressed in these transgenic mice. The increase in the reaction of several other proteins to this antibody might be due to post-translational processes of PGC-1α [33], its degradation products, or non-specific binding to unrelated proteins, although the precise nature of this is unknown.

In the transgenic mice, the expression of the PGC-1α target genes, COX2 and COX4, was also elevated in skeletal muscle but not in heart (Fig. 1A), confirming that expression of PGC-1α-b is specific to skeletal muscles.

Body weight, body composition and tissue weight were measured in male transgenic mice at 10 weeks of age. The body weight, lean body weight, fat weight, and fat% were not different between PGC-1α-b transgenic mice and wild-type littermates (Table 2). In PGC-1α-b transgenic mice, the weights of gastrocnemius, quadriceps, TA and extensor digitorum longus (EDL) were significantly lower than in wild-type littermates, however, this difference was not observed in the soleus.

**Table 2:** Body composition of male wild-type (Wt) and PGC-1α-b transgenic mice.

	Wt	PGC-1α-b	
		A-line	B-line
Body weight (g)	22.76±0.70	22.42±0.61	21.41±0.57
Lean (g)	18.61±0.43	18.33±0.41	18.87±0.54
Fat (g)	3.82±0.15	3.57±0.15	3.53±0.25
Fat (%)	17.07±0.44	16.33±0.38	15.73±0.88
Gas (g)	0.245±0.009	0.209±0.008**	0.210±0.006**
Qua (g)	0.294±0.012	0.240±0.009**	0.254±0.008*
TA (g)	0.085±0.003	0.088±0.004	0.077±0.002*
EDL (g)	0.018±0.001	0.016±0.000*	0.016±0.001*
Sol (g)	0.017±0.001	0.019±0.001	0.017±0.001

Values are means ± SE (n = 6–9 at 10 weeks of age).

*P<0.05,

**P<0.01 vs. Wt.

Gas, gastrocnemius; Qua, quadriceps; TA tibialis anterior; EDL, extensor digitorum longus; Sol, soleus.

**Figure 2:**
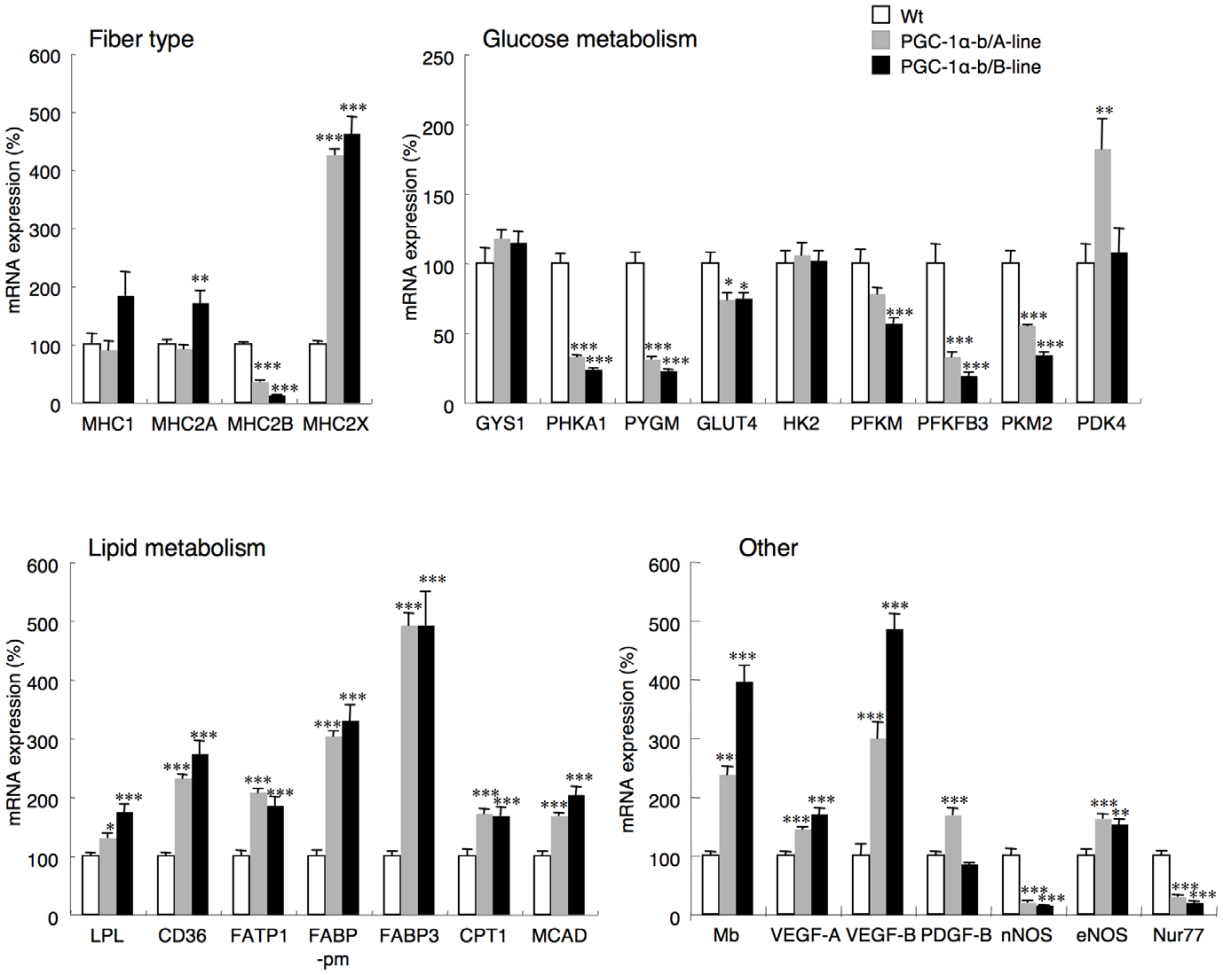
PGC-1α-b induced changes in gene expression in skeletal muscle. Results of quantitative RT-PCR analysis of transcripts encoding proteins involved in fiber type, glucose metabolism, lipid metabolism, and others in quadriceps from wild-type (Wt) and PGC-1α-b mice (A line and B line) at 8 weeks of age. Values are means ± SE (n = 6–9). * *P*<0.05, **P<0.01, *** *P*<0.001 vs. Wt. MHC, myosin heavy chain; GYS, glycogen synthase; PHKA, phosphorylase kinase alpha, PYGM, muscle glycogen phosphorylase; GLUT, glucose transporter; HK, hexokinase; PFKM, muscle phosphofructokinase; PFKFB, 6-phosphofructo-2-kinase/fructose-2,6-biphosphatase; PKM, muscle pyruvate kinase; PDK, pyruvate dehydrogenase kinase; LPL, lipoprotein lipase; FATP, fatty acid transport protein; FABP-pm, plasma membrane fatty acid binding protein; FABP, fatty acid binding protein; CPT, carnitine palmitoyltransferase; MCAD, medium chain acyl-CoA dehydrogenase; Mb, myoglobin; VEGF, vascular endothelial growth factor; PDGF, platelet-derived growth factor; nNOS, neuronal nitric oxide synthase; eNOS, endothelial nitric oxide synthase.

The expression of mRNA in skeletal muscles of genes related to muscle fiber type and metabolism was determined by quantitative real-time RT-PCR (Fig. 2). Expression of myosin heavy chain (MHC) 1 (slow-twitch oxidative fibers, rich in mitochondria) and 2A (fast-twitch oxidative fibers) in quadriceps was increased only in PGC-1α-b transgenic mice (line B), but the increase in MHC1 was not significantly different. Compared to wild-type littermates, expression of MHC 2B (fast-twitch glycolytic fibers) was decreased to 37% in line A and 13% in line B, and the expression of MHC 2X (mouse fast-twitch oxidative fibers) was increased to 426% in line A and 462% in line B PGC-1α-b transgenic mice. These data suggested that expression of oxidative fibers was increased and glycolytic fibers was decreased in PGC-1α-b transgenic mice, similar to MCK-PGC-1α-a transgenic mice [34]. The expression of genes involved in glycogenolysis, such as phosphorylase kinase alpha (PHKA) 1 and muscle glycogen phsphorylase (PYGM), were significantly decreased to 20–30% of wild-type in both lines of transgenic mice. Glucose transporter (GLUT) 4 was decreased to 75% in both lines of transgenic mice. The key enzymes for glycolysis, such as muscle phosphofructokinase (PFKM), 6-phosphofructo-2-kinase/fructose-2,6-biphosphatase (PFKFB) 3, and muscle pyruvate kinase (PKM) 2, were decreased significantly in the transgenic mice (the decrease of PFKM mRNA in line A transgenic mice was not significant), suggesting production of pyruvate was decreased in skeletal muscle that overexpressed PGC-1α-b. Pyruvate dehydrogenase kinase (PDK) 4 expression was increased only in line A transgenic mice. On the other hand, the expression of genes encoding proteins involved in fatty acid transport and fatty acid oxidation, such as lipoprotein lipase (LPL), CD36, fatty acid transport protein (FATP) 1, plasma membrane fatty acid binding protein (FABP-pm), fatty acid binding protein (FABP) 3, carnitine palmitoyltransferase (CPT) 1 and medium chain acyl-CoA dehydrogenase (MCAD), was higher in the transgenic mice. Myoglobin (Mb) and vascular endothelial growth factors (VEGFs) were also increased in the transgenic mice. Because VEGF-B might increase endothelial lipid uptake via stimulation of fatty acid transporter expression in endothelial cells [35], the increase in VEGF-B and fatty acid transporters in skeletal muscle overexpressing PGC-1α-b could enhance the rate of fatty acid transport from the blood stream into skeletal muscle. Neuronal nitric oxide synthase (nNOS) was decreased in the transgenic mice. Endothelial NOS (eNOS) was increased in the skeletal muscle overexpressing PGC-1α-b, suggesting that the numbers of endothelial cells derived from capillaries in the skeletal muscle tissue was increased. One of the NR4A orphan nuclear receptor proteins, Nur77, which regulates expression of genes linked to glucose metabolism [36], was significantly decreased in the transgenic mice.

### Mitochondrial number or volume in skeletal muscles increases, but the respiratory functions of mitochondria per se are normal in transgenic mice

**Figure 3:**
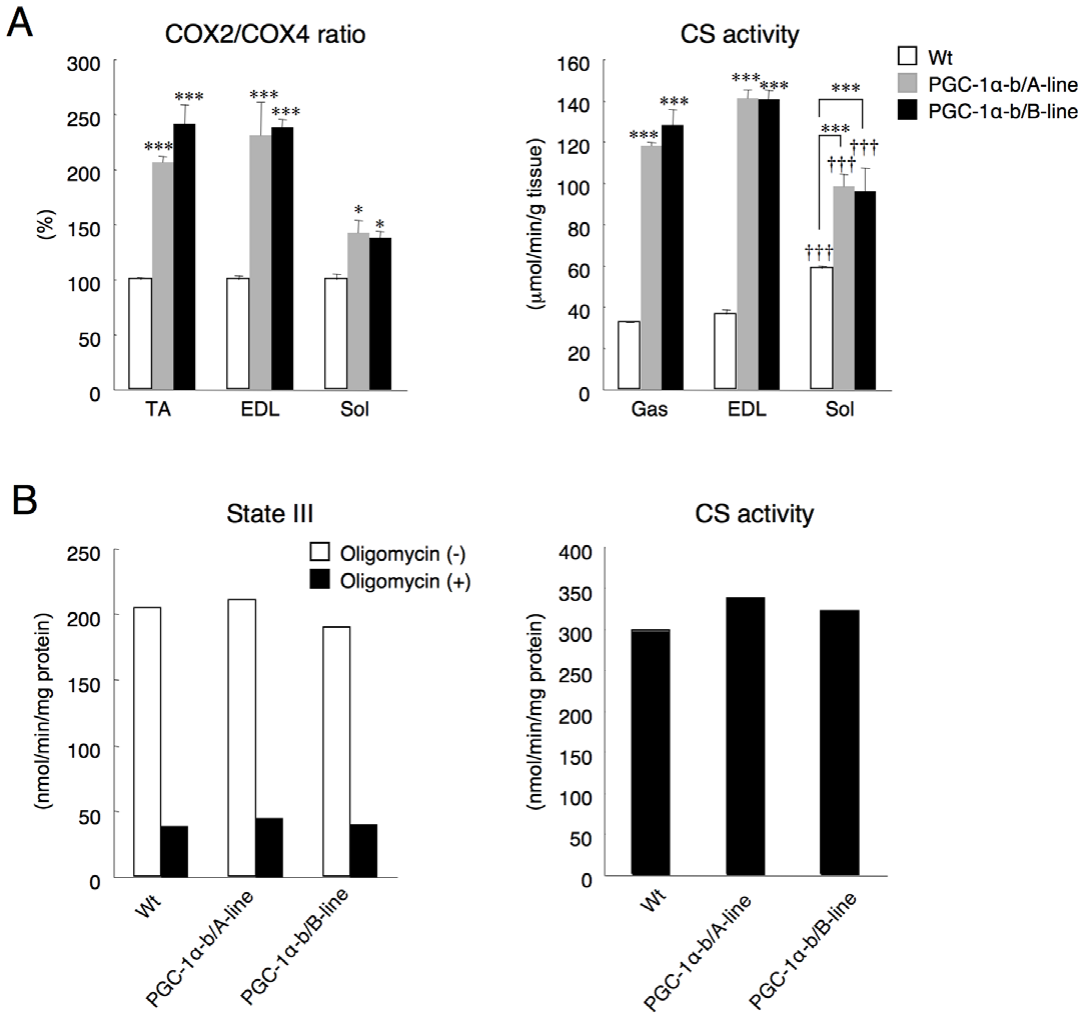
Mitochondrial volume and its function in skeletal muscles overexpressing PGC-1α-b. (A) For estimation of mitochondrial DNA copy number in skeletal muscle (tibialis anterior (TA), extensor digitorum longus (EDL), and soleus (Sol)), the relative mitochondrial DNA copy number from individual mice in each group was calculated as the ratio of COX2 (mitochondrial) to COX4 (nuclear) genes as determined by real-time PCR. The relative mitochondrial DNA copy number was expressed as the percentage of the ratio in wild-type (Wt). CS activity (gastrocnemius (Gas), EDL, and Sol) was normalized to tissue weight. Values are means ± SE of Wt and PGC-1α-b transgenic mice (A-line and B-line) at 10 weeks of age (n = 3–4). *P<0.05, ***P<0.001 vs. Wt. †††*P*<0.001 vs. Gas. (B) State III respiration rate of the mitochondrial fraction prepared from skeletal muscle (Gas, quadriceps, and TA) was measured in the presence and absence of 2.5 µg/ml oligomycin. CS activity was also measured in the mitochondrial fraction. Each data point is the mean value of the measurements normalized to the protein content of the fraction. Skeletal muscles were sampled from both Wt and PGC-1α-b transgenic mice (A-line and B-line) at 10 weeks of age. Oxidative phosphorylation of the mitochondrial fraction prepared from skeletal muscle was unchanged in mice overexpressing PGC-1α-b in skeletal muscle.

To estimate the numbers of mitochondria in the skeletal muscle from PGC-1α-b transgenic mice, the mitochondrial encoded-DNA (COX2) copy number relative to nuclear encoded-DNA (COX4) copy number and the CS activity were measured. The ratio of COX2 copy number to COX4 copy number in PGC-1α-b transgenic mice was higher than in wild-type mice (TA, 2.1-fold in line A and 2.4-fold in line B; EDL, 2.3-fold in line A and B; soleus, 1.4-fold in line A and B) (Fig. 3A). CS activity in the gastrocnemius (mixture of red and white fibers) and EDL (white fibers) of PGC-1α-b transgenic mice was increased 3.7- and 3.8-fold in line A and 4.0- and 3.8-fold in line B, respectively, relative to wild-type mice. Because CS activity of the soleus (red fibers) was 1.7-fold higher than those of the gastrocnemius and EDL in the wild-type, the activity in the soleus of PGC-1α-b transgenic mice was increased only 1.7-fold in line A and 1.6-fold in line B relative to wild-type mice. These data suggested that over-expression of PGC-1α-b in skeletal muscle increased the biogenesis of mitochondria and that this increase was larger in white glycolytic muscle than red oxidative muscle.

To study the function of the skeletal muscle mitochondria themselves, we compared the respiration rates ( = oxygen consumption) in isolated mitochondria from gastrocnemius, quadriceps, and TA of PGC-1α-b transgenic mice with those in wild-type mice. Both lines of PGC-1α-b transgenic mice showed almost the same respiration rate as wild-type on a protein basis of a mitochondria-rich fraction under conditions where the mitochondrial substrate and ADP were not limiting (Fig. 3B), which mimicked state III respiration [37]. To measure uncoupled respiration, oligomycin, an inhibitor of the F_1_F_0_-ATP synthase, was added to inhibit oxidative phosphorylation prior to measurement of respiration rates. Oligomycin-insensitive respiration ( = uncoupling) constituted about 20% of the total respiration in wild-type and PGC-1α-b transgenic mice. CS activity in the isolated mitochondrial fraction was not different between wild-type and PGC-1α-b transgenic mice.

These data suggested that the number of mitochondria was increased in skeletal muscle from PGC-1α-b transgenic mice and that the mitochondrial oxidative phosphorylation was the same as in wild-type.

### Increased capillary number in transgenic mice

**Figure 4:**
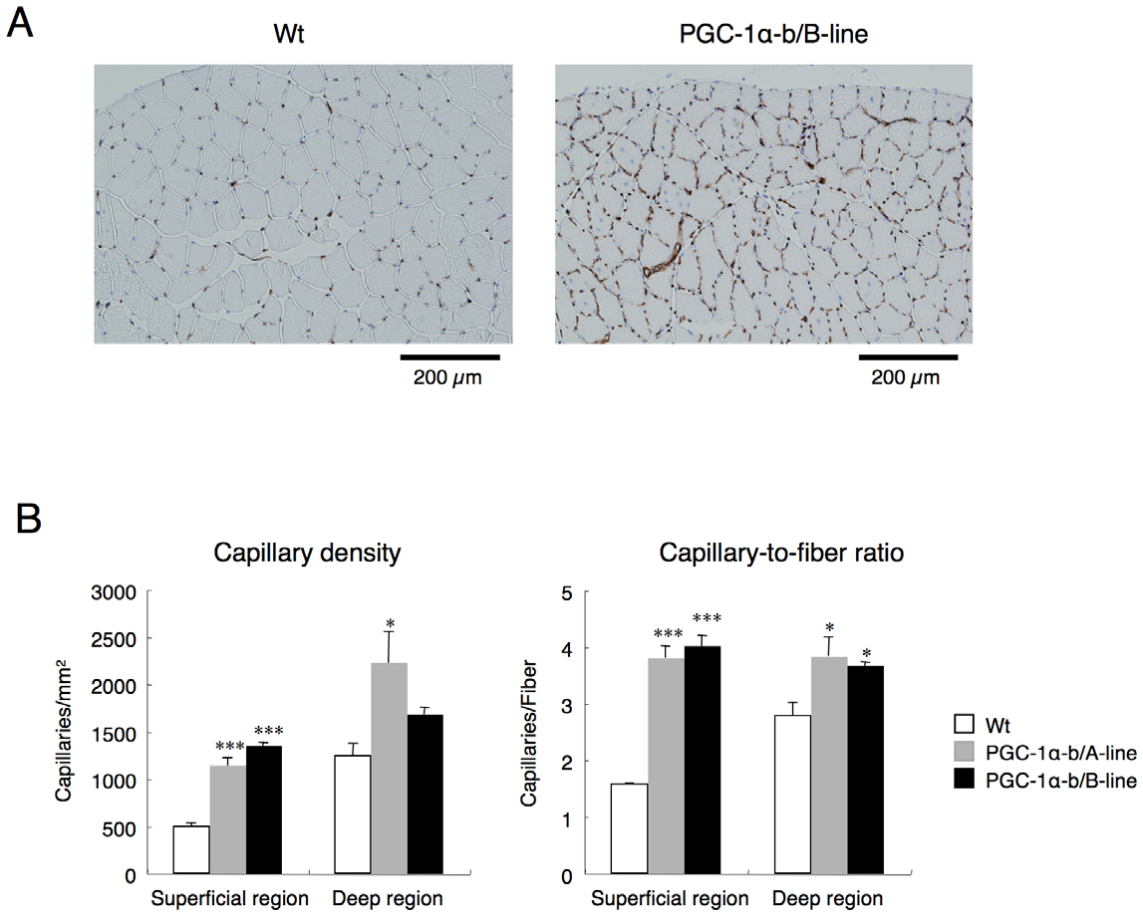
Skeletal muscle-specific expression of PGC-1α-b induced angiogenesis in skeletal muscle. (A) Transverse frozen sections of TA from wild-type (Wt) and PGC-1α-b transgenic mice at 10–11 weeks of age were immunostained for CD31 (endothelial-specific PECAM). Representative immunostains in the superficial region is shown. (B) Quantification of CD31-positive capillaries/mm^2^ and capillaries per individual myofiber in superficial and deep regions (n = 3 per group). Data are presented as mean ± SE. *, *P*<0.05, ****, *P*<0.001 vs. Wt.

Exercise promotes angiogenesis [38]–[42]. PGC-1α-b might be a candidate for inducing angiogenic processes because transfection of PGC-1α-b into differentiated primary skeletal myocytes isolated from mice increased VEGF and PDGF-B mRNA expression [12]. This causal relationship was also observed *in vivo*. Overexpression of PGC-1α-b in skeletal muscle increased VEGF-A mRNA expression (Fig. 2). Furthermore, immunohistochemical analysis of TA muscle was performed to investigate whether PGC-1α-b induces angiogenesis *in vivo* (Fig. 4). Each section was analyzed in superficial and deep regions, and 209–774 and 476–1670 of CD31 positive capillaries were identified in each photograph of the superficial and deep regions, respectively. The capillary-to-fiber ratio was increased 2.4–2.6 and 1.3–1.4 fold in the superficial and deep regions of PGC-1α-b transgenic mice, respectively. Because the superficial region in TA muscle from wild-type mice was rich in white glycolytic fibers and had a lower capillary density, it was suggested that PGC-1α-b-induced angiogenesis in vivo occurred especially in regions where white glycolytic fibers changed to red oxidative fibers.

### Substrate utilization is not altered in the sedentary and fasting state in transgenic mice

Increases in mitochondria and capillary numbers in skeletal muscles in transgenic mice may alter substrate utilization. To examine this possibility, 8 week-old males of both sedentary PGC-1α-b transgenic mice and wild-type littermates were subjected to fasting and their oxygen consumption measured and RQ ratio calculated. The oxygen consumption and RQ ratio for PGC-1α-b transgenic and wild-type mice did not differ during the dark cycle (feeding period) or light cycle (sleeping period) (Table 3). Daily activity levels were not different between PGC-1α-b transgenic mice and wild-type littermates. Body weight was similarly reduced in the two groups after 24-hours of fasting (if energy expenditure was enhanced, fasting-induced weight reduction increased), suggesting that over-expression of PGC-1α-b in skeletal muscles did not affect fatty acid oxidation during fasting.

**Table 3:** Substrate utilization is not altered in the sedentary and fasting states in transgenic mice.

		Wt	PGC-1α-b
Dark cycle	VO_2_ (ml/min/kg^0.75^)	19.0±0.3	19.8±0.5
	VCO_2_ (ml/min/kg^0.75^)	13.7±0.2	14.4±0.4
	RQ ratio	0.71±0.00	0.72±0.00
	Glucose oxidation (mg/min/kg^0.75^)	1.27±0.40	1.95±0.19
	Lipid oxidation (mg/min/kg^0.75^)	8.9±0.2	9.0±0.2
	Energy production (cal/min/kg^0.75^)	89.4±1.3	93.5±2.3
	Total spontaneous physical activity (count)	64873±3168	56807±6414
Light cycle	VO_2_ (ml/min/kg^0.75^)	13.9±0.4	13.9±0.2
	VCO_2_ (ml/min/kg^0.75^)	9.9±0.3	10.0±0.1
	RQ ratio	0.71±0.00	0.72±0.00
	Glucose oxidation (mg/min/kg^0.75^)	0.32±0.28	0.91±0.15
	Lipid oxidation (mg/min/kg^0.75^)	6.7±0.1	6.5±0.1
	Energy production (cal/min/kg^0.75^)	65.1±1.7	65.3±0.7
	Total spontaneous physical activity (count)	9542±1741	9069±1269
Body weight (g)	Before fasting	25.0±0.6	24.4±0.4
	After fasting	21.3±0.4	20.8±0.4
	Difference during fasting	−3.7±0.2	−3.7±0.1

Oxygen consumption (VO_2_), carbon dioxide production (VCO_2_), RQ ratio, spontaneous physical activity, calculated glucose and lipid oxidation rate, and energy production rate were measured while mice were fasted for 24 hours. Male wild-type (Wt) and PGC-1α-b transgenic mice (line A) at 8 weeks of age. Values are means ± SE (n = 6–8). Significant differences were not observed.

These data suggested that increases in mitochondria and capillaries in skeletal muscles did not affect substrate utilization in the sedentary state.

### Exercise capacity of PGC-1α-b transgenic mice and wild-type littermates

**Figure 5:**
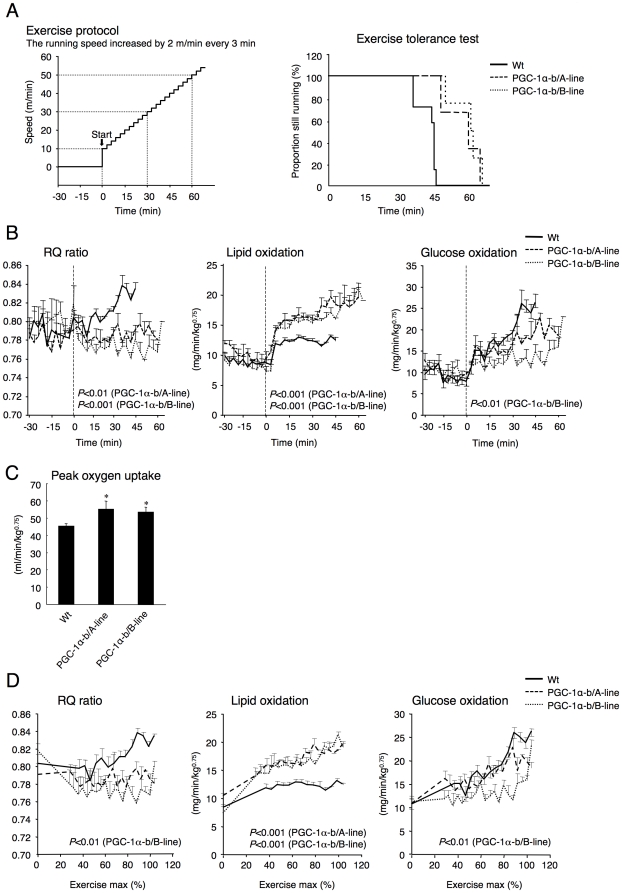
Exercise tolerance, RQ ratio, and calculated glucose and lipid oxidation rate, during exercise. (A) PGC-1α-b transgenic mice (A-line and B-line, n = 3 and 4) and wild-type littermates (Wt, n = 7) (each 8 weeks old) were exercised by forced running on a treadmill at 10 m/min. The speed increased by 2 m/min every 3 min until exhaustion. Mice ran until exhaustion (exercise tolerance test). Exercise tolerance is shown as a Kaplan-Meier survival curve. A significant difference (*P*<0.01, log-rank test) was observed between the exercise tolerances of both lines of PGC-1α-b transgenic mice vs. Wt. (B) Oxygen consumption and carbon dioxide production were monitored using an O_2_/CO_2_ metabolism measuring system for small animals, which was equipped with an air-tight treadmill chamber. The RQ ratio, and calculated glucose and lipid oxidation rates are shown. Each value is the mean ± SE of 3–7 mice. Statistical significance was calculated as the area under the curve when sedentary and during running (0–36 min). Significant differences were not observed when sedentary for any parameters. *P* values vs. Wt are shown in the figure in cases where the difference is significant. (C) Peak oxygen uptake as defined by the measurement of oxygen consumption at the point of failure, is shown. *P<0.05 vs. Wt. (D) The RQ ratio, and calculated glucose and lipid oxidation rate are plotted against relative exercise intensity, as estimated by the percentage of mean speed at exhaustion for each genotype. Statistical significance was calculated as the area under the curve using the mean value at rest for each genotype as base line. *P* values vs. Wt are shown in the figure in cases where the difference is significant.

The ability of PGC-1α-b transgenic mice to tolerate a bout of exercise might be altered. To examine this possibility, mice were started off running on a treadmill at 10 m/min, and then the speed was increased by 2 m/min every 3 min until exhaustion (Fig. 5A). PGC-1α-b transgenic mice could run for a significantly longer duration with higher exercise-intensity than wild-type littermates (*P* = 0.002), although the weights of gastrocnemius, quadriceps, TA and EDL were significantly lower than in wild-type littermates (Table 2). Wild-type littermates ran for a duration of 47 min at a maximum speed of 40 m/min (total distance was 1.2 km), whereas PGC-1α-b transgenic mice ran for a duration of 64 and 66 min at a maximum speed of 54 and 56 m/min in lines A and B (total distances were 2.2 or 2.4 km), respectively (Fig. 5A).

To investigate the fuel utilization during exercise and the peak oxygen consumption, VO_2_ and VCO_2_ were monitored simultaneously until mice were exhausted. VO_2_ and VCO_2_ were increased gradually as speed increased until exhaustion in every group of mice (data not shown). In wild-type mice, the RQ ratio during exercise, which indicated fuel utilization, was increased as the treadmill speed increase, suggesting that glucose oxidation became predominant with increasing exercise intensity (Fig. 5B). However, in both lines of PGC-1α-b transgenic mice, an increase in the RQ ratio and glucose oxidation with increasing treadmill speed was not observed. The calculated level of lipid oxidation during exercise was always higher in PGC-1α-b transgenic mice than wild-type mice, suggesting that lipid oxidation was the predominant fuel source for exercise in the transgenic mice. Both lines of transgenic mice demonstrated a 20% increase in their peak oxygen uptake compared to wild-type mice (Fig. 5C).

Because of the difference in maximum running speed between wild-type and transgenic mice, it is possible that increased fatty acid oxidation in transgenic mice was due to their increased exercise capacity. However, when the data are expressed as the percentage of maximal speed, PGC-1α-b transgenic mice still showed a lower RQ ratio and higher levels of calculated fat oxidation (Fig. 5D).

These data indicated that PGC-1α-b transgenic mice show increased fat oxidation and decreased carbohydrate oxidation at either the absolute exercise intensity, or the same relative exercise intensities.

### Decrease in consumption of carbohydrate as fuel during exercise in PGC-1α-b transgenic mice

**Figure 6:**
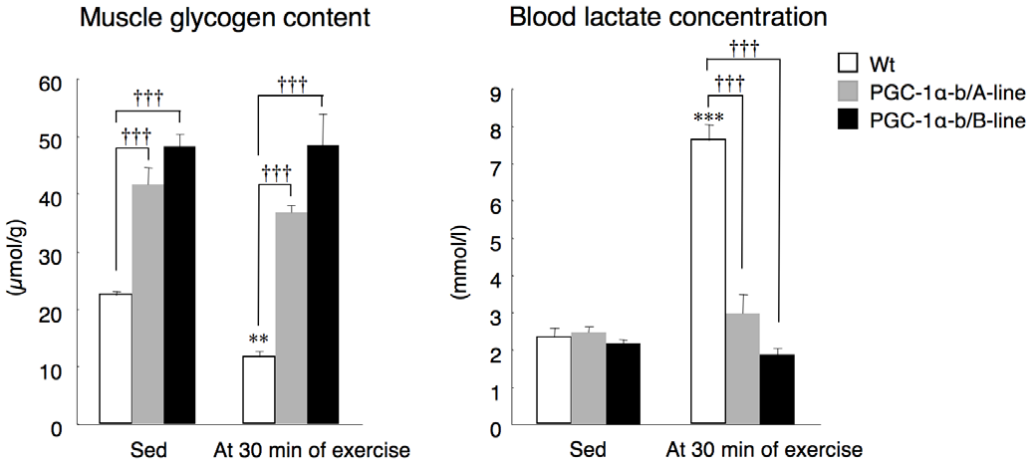
Skeletal muscle glycogen content and blood lactate concentration before and after exercise. PGC-1α-b transgenic mice and wild-type littermates (Wt) (each 8 weeks old) were exercised by forced running on a treadmill at 10 m/min. The speed increased 2 m/min every 3 min up to 30 m/min. Mice ran for 30 min. Skeletal muscle (gastrocnemius) glycogen content was measured before (Sed) and after 30 min of exercise (n = 3–5). Values are means ± SE. **P<0.01 vs. Sed. †††*P*<0.001 vs. Wt. Blood lactate concentration was measured before (Sed) and at 30 min of exercise (n = 4–6). Values are means ± SE. ***P<0.001 vs. Sed. †††*P*<0.001 vs. Wt.

Lipid oxidation was the predominant fuel source for exercise in PGC-1α-b transgenic mice, whereas consumption of carbohydrate during exercise was reduced. Glycogen concentration in skeletal muscles and blood lactate concentration were measured at rest and 30 min after the exercise tolerance test, a time when the shift of fuel usage to glucose was manifested. At rest, the glycogen content in the gastrocnemius in line A and line B PGC-1α-b transgenic mice was 1.9- and 2.2-fold greater than in wild-type mice (Fig. 6). At the 30 min mark of the exercise tolerance test, skeletal muscle glycogen content was decreased to 52% in wild-type mice but no significant decrease was observed in transgenic mice. The liver glycogen content was not significantly changed between rest and 30 min of exercise in either wild-type or transgenic mice (data not shown). The blood lactate concentration at 30 min from the start of exercise was increased in wild-type mice, however this increase was not observed in transgenic mice (Fig. 6). Furthermore, an increase in blood lactate concentration at exhaustion was not observed in the transgenic mice (data not shown), also suggesting that glycogen in skeletal muscle was not used preferentially during exercise in transgenic mice. The blood glucose concentration did not differ significantly between the two groups of mice (data not shown).

## Discussion

**Figure 7:**
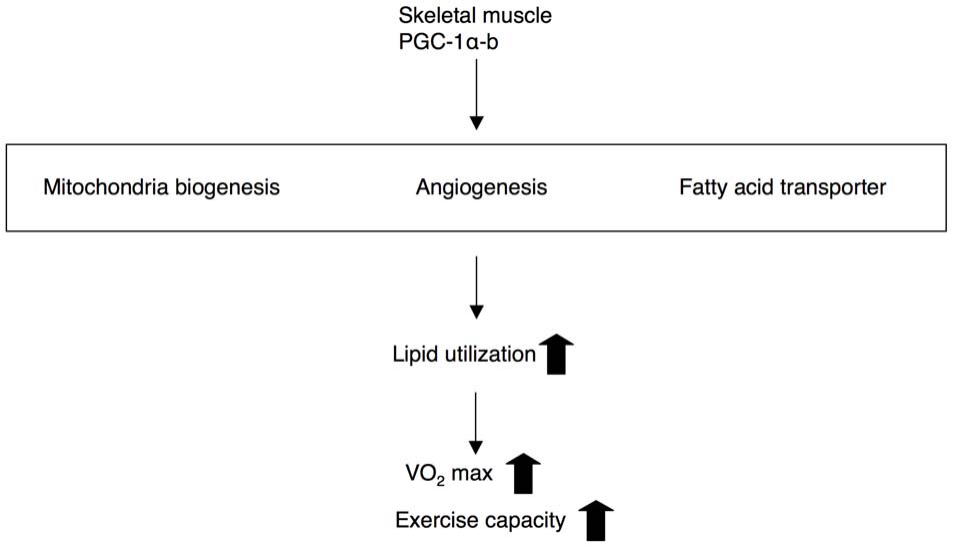
PGC-1α-b-mediated increase in fatty acid utilization in skeletal muscle improves maximal oxygen uptake and exercise capacity.

In this study, we created mice that overexpressed PGC-1α-b in skeletal muscle but not in heart. PGC-1α-b protein, whose amino terminus is different from that of PGC-1α-a protein, is a predominant PGC-1α isoform in skeletal muscles that is expressed in response to exercise. This is the first *in vivo* study to examine exercise capacity in mice overexpressing PGC-1α-b in skeletal muscle. The alterations in the phenotypes of our transgenic mice were comparable with those induced by exercise training in mice and humans. CS activity was increased 3-fold in our transgenic mice, whereas rats showed a 2-fold increase in mitochondrial enzymes and in the capacity of skeletal muscle to oxidize pyruvate, after 12 weeks of treadmill exercise training [43]. The 20% increase in peak oxygen uptake in transgenic mice was comparable with the 9–19% increase in VO_2max_ observed after exercise training in humans [5]. Our data indicated that alterations in metabolism of skeletal muscles (increased mitochondrial biogenesis, angiogenesis, and fatty acid transporter) also contribute to whole body VO_2max_ and exercise capacity (Fig. 7), in addition to the contribution of the pump capacity of the heart.

Fat oxidation rates increase from low- to moderate-intensities and then decrease when the intensity becomes high due to reduced fatty acid availability and reduced activity of CPT1 [44]. It is also well known that endurance training can increase fat oxidation during submaximal exercise [44]–[49]. Maximal rates of fat oxidation were shown to be reached at intensities between 59% and 64% of maximum oxygen consumption in trained individuals and between 47% and 52% of maximum oxygen consumption in a large sample of the general population [44]. Increases in fat oxidation with exercise training were reported when trained and untrained individuals were compared at the same absolute and relative exercise intensities [50]. Similar to human athletes, mice over-expressing PGC-1α-b showed increased fat oxidation at the same absolute and relative exercise intensities. Increased fat oxidation during exercise could provide sufficient energy for muscle contraction and contribute to improved endurance capacity and exercise performance, as suggested in a previous study [51].

As for mechanisms of increased fatty acid utilization in skeletal muscles during exercise in PGC-1α-b transgenic mice, increases in expression of enzymes for fatty acid β oxidation (MCAD, CPT1 etc.) via activation of PPARα [13], and mitochondrial capacity via activation of nuclear respiratory factor 1(NRF-1) and NRF-2 [52] contribute to increased fatty acid oxidation. An increase in fatty acid transport may also contribute to fatty acid utilization. Expression of the fatty acid transporter was increased 2 to 5-fold in skeletal muscle overexpressing PGC-1α-b. It was reported that the promoter region of CD36 and FATP1 contains a peroxisome proliferator-responsive element (PPRE) [53], [54]. The presence of a PPRE in the promoter of FABP-pm was not reported, but is likely as PPARγ activation induced the expression of FABP-pm in rat skeletal muscle [55]. These findings suggest that PGC-1α-b activates expression of the fatty acid transporter via co-activation of PPARs. Recently, it was shown that VEGF-B was tightly co-expressed with nuclear-encoded mitochondrial genes and specifically controlled endothelial uptake of fatty acids via transcriptional regulation of vascular fatty acid transport proteins [35]. Expression of VEGF-B was probably activated via activation of estrogen-related receptorα (ERRα) and its co-activator PGC-1α [35], [56]. In the skeletal muscle overexpressing PGC-1α-b, an increase in the expression of VEGF-B might enhance endothelial uptake of fatty acids. Stimulation of angiogenesis could also contribute to increased fatty acid oxidation because of the increase in substrate delivery into the skeletal muscle. PGC-1α-b plays a role in exercise-induced angiogenesis in skeletal muscle [12]. The β-AR mediated induction of PGC-1α-b stimulates a broad program of angiogenic factors, including VEGF, via activation of ERRα. In fact, skeletal muscle VEGF-A expression and the number of capillaries were significantly increased in PGC-1α-b transgenic mice.

Regarding mechanisms of decreased carbohydrate utilization in skeletal muscles during exercise in PGC-1α-b transgenic mice, inhibition of glycogenolysis and glycolysis was indicated. There was no increase in blood lactate concentration in PGC-1α-b transgenic mice at exhaustion, suggesting that muscle glycogen usage was absent in the transgenic mice as reported by Wende et al. [57]. Inhibition of muscle glycogen usage was probably due to a decrease in expression of genes related to glucose metabolism following overexpression of PGC-1α-b in the skeletal muscle. The expression of enzymes for glucose metabolism, such as PHKA1, PYGM, GLUT4, PFKM, was mediated by one of the NR4A orphan nuclear receptors, Nur77 [36]. In PGC-1α-b transgenic mice, reduction of Nur77 mRNA levels in skeletal muscle was observed concomitantly with a decrease in the expression of genes linked to glucose metabolism. The mechanism for the decrease in Nur77 expression in response to overexpression of PGC-1α-b is unclear at this time. However, because the level of Nur77 mRNA was lower in soleus muscles (predominantly type 1, slow-twitch, oxidative fibers) than fast-twitch glycolytic muscles [36], PGC-1α-b mediated change in the muscle fiber type might be associated with the decrease in Nur77 expression. Taken together, these data suggest that PGC-1α-b stimulates fatty acid oxidation and inhibits carbohydrate oxidation simultaneously via broad actions as a co-activator of transcription factors.

In the case of PGC-1α-a, another isoform that was studied extensively [11], [33], there were two studies to examine its effects on exercise performance but their results were inconsistent [16], [57]. Initially, Wende et al. reported that conditional induction of PGC-1α-a in skeletal muscle (not in heart) for 3–4 wk showed increases in mitochondrial biogenesis, the capacity for mitochondrial fatty acid oxidation, and muscle glycogen stores [57]. Exercise performance was not different between control (4.2 km) and conditional PGC-1α-a mice (3.3 km) under a low-intensity exercise protocol. However, these conditional PGC-1α-a mice could not tolerate a high-intensity exercise protocol; control mice could run 1.7 km and PGC-1α-a mice could run only 0.8 km, possibly due to the inability to use muscle glycogen during exercise. In this study, intensities at exhaustion were 80 m/min for control and 65 m/min for PGC-1α-a mice, suggesting that conditional over-expression of PGC-1α-a did not have an affect on endurance performance but reduced performance under extremely high-intensity exercise. In contrast, Calvo et al. reported that PGC-1α-a transgenic mice, in which PGC-1α-a expression was increased both in skeletal muscle and heart constitutively, showed increased fatty acid oxidation and decreased glycogen usage during exercise, but also improved exercise performance and peak oxygen uptake [16]. In their study, PGC-1α-a transgenic mice could run 2.0 km and wild-type mice could run 1.2 km under a low-intensity exercise protocol (*p*<0.001). The transgenic mice could also tolerate more high-intensity exercise than wild-type mice (*p*<0.001). Because these two studies used different exercise protocols, both for the low-intensity (different duration at initial speed and incline) and high-intensity (intermittent or continuous protocols) levels, it is difficult to compare the exercise capacity in mice between these studies. Although we used different protocols (high-intensity and continuous protocols without an incline) to determine the exercise capacity, our PGC-1α-b mice could tolerate a higher intensity of exercise than wild-type mice, which is similar to the results observed in Calvo's study for PGC-1α-a transgenic mice and their higher exercise performance than wild-type mice.

There are several genetic mouse models that show increased exercise performance, however their skeletal muscle glycogen content is variable. Overexpression of constitutive active calcineurin in skeletal muscle resulted in increased endurance performance and mitochondrial function with a higher glycogen content in skeletal muscles [58]. Mice deficient in actinin-3, in which expression was restricted largely to the fast glycolytic skeletal muscles fibers, showed a more efficient aerobic pathway and an increase in intrinsic endurance performance with a higher glycogen content [59], [60]. However, overexpression of a cytosolic form of phosphoenolpyruvate carboxykinase in skeletal muscle also enhanced exercise capacity, and increased in mitochondrial biogenesis and fatty acid utilization during endurance exercise, but with a lower glycogen content [61]. This model mouse relied heavily on fatty acids as a source of muscle energy during exercise and did not use carbohydrate because no lactate generation was observed at exhaustion. In contrast, a mouse model with increased glycolysis and glycogen content in skeletal muscles showed a decreased running capacity [62]. A conditional transgenic mouse expressing a constitutively active form of Akt1 showed muscle hypertrophy due to the growth of type IIb muscle fibers (glycogen rich fibers), which was accompanied by an increase in strength, but showed a reduced capacity for running. These studies suggest that although inhibition of muscle glycogen usage may decrease high intensity exercise (possibly a model of resistance training) as observed in the conditional PGC-1α-a induction model, a shift in the skeletal muscle phenotype to oxidative metabolism and its increase in lipid utilization during exercise contributes largely to endurance performance.

In conclusion, a PGC-1α-b-mediated increase in mitochondrial biogenesis and capillary density in skeletal muscles contributes to improved exercise capacity. Adaptation to exercise training is partly due to the induction and activation of PGC-1α-b. Increases in PGC-1α-b protein or function might be a useful strategy for sedentary subjects to perform exercise efficiently, and this may aid in prevention of life-style related diseases and lead to an increased lifespan.

## References

[1] Lee DC, Artero EG, Sui X, Blair SN (2010). Mortality trends in the general population: the importance of cardiorespiratory fitness.. J Psychopharmacol.

[2] Lee DC, Sui X, Ortega FB, Kim YS, Church TS (2011). Comparisons of leisure-time physical activity and cardiorespiratory fitness as predictors of all-cause mortality in men and women.. Br J Sports Med.

[3] Kodama S, Saito K, Tanaka S, Maki M, Yachi Y (2009). Cardiorespiratory fitness as a quantitative predictor of all-cause mortality and cardiovascular events in healthy men and women: a meta-analysis.. JAMA.

[4] Bouchard C, Daw EW, Rice T, Perusse L, Gagnon J (1998). Familial resemblance for VO2max in the sedentary state: the HERITAGE family study.. Med Sci Sports Exerc.

[5] Bouchard C, Sarzynski MA, Rice TK, Kraus WE, Church TS (2011). Genomic predictors of the maximal O uptake response to standardized exercise training programs.. J Appl Physiol.

[6] Saltin B, Calbet JA (2006). Point: in health and in a normoxic environment, VO2 max is limited primarily by cardiac output and locomotor muscle blood flow.. J Appl Physiol.

[7] Suter E, Hoppeler H, Claassen H, Billeter R, Aebi U (1995). Ultrastructural modification of human skeletal muscle tissue with 6-month moderate-intensity exercise training.. Int J Sports Med.

[8] Tonkonogi M, Sahlin K (1997). Rate of oxidative phosphorylation in isolated mitochondria from human skeletal muscle: effect of training status.. Acta Physiol Scand.

[9] Tjonna AE, Lee SJ, Rognmo O, Stolen TO, Bye A (2008). Aerobic interval training versus continuous moderate exercise as a treatment for the metabolic syndrome: a pilot study.. Circulation.

[10] Wisloff U, Stoylen A, Loennechen JP, Bruvold M, Rognmo O (2007). Superior cardiovascular effect of aerobic interval training versus moderate continuous training in heart failure patients: a randomized study.. Circulation.

[11] Puigserver P, Wu Z, Park CW, Graves R, Wright M (1998). A cold-inducible coactivator of nuclear receptors linked to adaptive thermogenesis.. Cell.

[12] Chinsomboon J, Ruas J, Gupta RK, Thom R, Shoag J (2009). The transcriptional coactivator PGC-1alpha mediates exercise-induced angiogenesis in skeletal muscle.. Proc Natl Acad Sci U S A.

[13] Miura S, Kai Y, Kamei Y, Ezaki O (2008). Isoform-specific increases in murine skeletal muscle peroxisome proliferator-activated receptor-gamma coactivator-1alpha (PGC-1alpha) mRNA in response to beta2-adrenergic receptor activation and exercise.. Endocrinology.

[14] Yoshioka T, Inagaki K, Noguchi T, Sakai M, Ogawa W (2009). Identification and characterization of an alternative promoter of the human PGC-1alpha gene.. Biochem Biophys Res Commun.

[15] Tadaishi M, Miura S, Kai Y, Kawasaki E, Koshinaka K (2011). Effect of exercise intensity and AICAR on isoform-specific expressions of murine skeletal muscle PGC-1alpha mRNA: a role of beta-adrenergic receptor activation.. Am J Physiol Endocrinol Metab.

[16] Calvo JA, Daniels TG, Wang X, Paul A, Lin J (2008). Muscle-specific expression of PPARgamma coactivator-1alpha improves exercise performance and increases peak oxygen uptake.. J Appl Physiol.

[17] Nagy TR, Clair AL (2000). Precision and accuracy of dual-energy X-ray absorptiometry for determining in vivo body composition of mice.. Obes Res.

[18] Miura S, Tomitsuka E, Kamei Y, Yamazaki T, Kai Y (2006). Overexpression of peroxisome proliferator-activated receptor gamma co-activator-1alpha leads to muscle atrophy with depletion of ATP.. Am J Pathol.

[19] Srere PA (1969). Citrate synthase.. Methods Enzymol.

[20] Bruce CR, Brolin C, Turner N, Cleasby ME, van der Leij FR (2007). Overexpression of carnitine palmitoyltransferase I in skeletal muscle in vivo increases fatty acid oxidation and reduces triacylglycerol esterification.. Am J Physiol Endocrinol Metab.

[21] Bruce CR, Thrush AB, Mertz VA, Bezaire V, Chabowski A (2006). Endurance training in obese humans improves glucose tolerance and mitochondrial fatty acid oxidation and alters muscle lipid content.. Am J Physiol Endocrinol Metab.

[22] Cogswell AM, Stevens RJ, Hood DA (1993). Properties of skeletal muscle mitochondria isolated from subsarcolemmal and intermyofibrillar regions.. Am J Physiol.

[23] Trounce IA, Kim YL, Jun AS, Wallace DC (1996). Assessment of mitochondrial oxidative phosphorylation in patient muscle biopsies, lymphoblasts, and transmitochondrial cell lines.. Methods Enzymol.

[24] Brey EM, King TW, Johnston C, McIntire LV, Reece GP (2002). A technique for quantitative three-dimensional analysis of microvascular structure.. Microvasc Res.

[25] Horak ER, Leek R, Klenk N, LeJeune S, Smith K (1992). Angiogenesis, assessed by platelet/endothelial cell adhesion molecule antibodies, as indicator of node metastases and survival in breast cancer.. Lancet.

[26] Davis JM, Kohut ML, Colbert LH, Jackson DA, Ghaffar A (1997). Exercise, alveolar macrophage function, and susceptibility to respiratory infection.. J Appl Physiol.

[27] Davis JM, Weaver JA, Kohut ML, Colbert LH, Ghaffar A (1998). Immune system activation and fatigue during treadmill running: role of interferon.. Med Sci Sports Exerc.

[28] Ferrannini E (1988). The theoretical bases of indirect calorimetry: a review.. Metabolism.

[29] Weir JB (1949). New methods for calculating metabolic rate with special reference to protein metabolism.. J Physiol.

[30] Lowry OH, Passonneau JV (1972). A Flexible System of Enzymatic Analysis.

[31] Aquilano K, Vigilanza P, Baldelli S, Pagliei B, Rotilio G (2010). Peroxisome proliferator-activated receptor gamma co-activator 1alpha (PGC-1alpha) and sirtuin 1 (SIRT1) reside in mitochondria: possible direct function in mitochondrial biogenesis.. J Biol Chem.

[32] Baar K, Wende AR, Jones TE, Marison M, Nolte LA (2002). Adaptations of skeletal muscle to exercise: rapid increase in the transcriptional coactivator PGC-1.. FASEB J.

[33] Handschin C, Spiegelman BM (2006). Peroxisome proliferator-activated receptor gamma coactivator 1 coactivators, energy homeostasis, and metabolism.. Endocr Rev.

[34] Arany Z, Lebrasseur N, Morris C, Smith E, Yang W (2007). The transcriptional coactivator PGC-1beta drives the formation of oxidative type IIX fibers in skeletal muscle.. Cell Metab.

[35] Hagberg CE, Falkevall A, Wang X, Larsson E, Huusko J (2010). Vascular endothelial growth factor B controls endothelial fatty acid uptake.. Nature.

[36] Chao LC, Zhang Z, Pei L, Saito T, Tontonoz P (2007). Nur77 coordinately regulates expression of genes linked to glucose metabolism in skeletal muscle.. Mol Endocrinol.

[37] Brand M, Brown G, Cooper C (1995). Mitochondria: A Practical Approach.;.

[38] Arany Z, Foo SY, Ma Y, Ruas JL, Bommi-Reddy A (2008). HIF-independent regulation of VEGF and angiogenesis by the transcriptional coactivator PGC-1alpha.. Nature.

[39] Bloor CM (2005). Angiogenesis during exercise and training.. Angiogenesis.

[40] Egginton S (2009). Invited review: activity-induced angiogenesis.. Pflugers Arch.

[41] Jain RK (2003). Molecular regulation of vessel maturation.. Nat Med.

[42] Prior BM, Yang HT, Terjung RL (2004). What makes vessels grow with exercise training?. J Appl Physiol.

[43] Holloszy JO (1967). Biochemical adaptations in muscle. Effects of exercise on mitochondrial oxygen uptake and respiratory enzyme activity in skeletal muscle.. J Biol Chem.

[44] Achten J, Jeukendrup AE (2004). Optimizing fat oxidation through exercise and diet.. Nutrition.

[45] Jansson E, Kaijser L (1987). Substrate utilization and enzymes in skeletal muscle of extremely endurance-trained men.. J Appl Physiol.

[46] Klein S, Coyle EF, Wolfe RR (1994). Fat metabolism during low-intensity exercise in endurance-trained and untrained men.. Am J Physiol.

[47] Sidossis LS, Wolfe RR, Coggan AR (1998). Regulation of fatty acid oxidation in untrained vs. trained men during exercise.. Am J Physiol.

[48] Turcotte LP, Richter EA, Kiens B (1992). Increased plasma FFA uptake and oxidation during prolonged exercise in trained vs. untrained humans.. Am J Physiol.

[49] Coggan AR, Raguso CA, Gastaldelli A, Sidossis LS, Yeckel CW (2000). Fat metabolism during high-intensity exercise in endurance-trained and untrained men.. Metabolism.

[50] Friedlander AL, Casazza GA, Horning MA, Buddinger TF, Brooks GA (1998). Effects of exercise intensity and training on lipid metabolism in young women.. Am J Physiol.

[51] Hawley JA, Brouns F, Jeukendrup A (1998). Strategies to enhance fat utilisation during exercise.. Sports Med.

[52] Scarpulla RC (1996). Nuclear respiratory factors and the pathways of nuclear-mitochondrial interaction.. Trends Cardiovasc Med.

[53] Frohnert BI, Hui TY, Bernlohr DA (1999). Identification of a functional peroxisome proliferator-responsive element in the murine fatty acid transport protein gene.. J Biol Chem.

[54] Tontonoz P, Nagy L, Alvarez JG, Thomazy VA, Evans RM (1998). PPARgamma promotes monocyte/macrophage differentiation and uptake of oxidized LDL.. Cell.

[55] Benton CR, Koonen DP, Calles-Escandon J, Tandon NN, Glatz JF (2006). Differential effects of contraction and PPAR agonists on the expression of fatty acid transporters in rat skeletal muscle.. J Physiol.

[56] Muoio DM (2010). Metabolism and vascular fatty acid transport.. N Engl J Med.

[57] Wende AR, Schaeffer PJ, Parker GJ, Zechner C, Han DH (2007). A role for the transcriptional coactivator PGC-1alpha in muscle refueling.. J Biol Chem.

[58] Jiang LQ, Garcia-Roves PM, de Castro Barbosa T, Zierath JR (2010). Constitutively active calcineurin in skeletal muscle increases endurance performance and mitochondrial respiratory capacity.. Am J Physiol Endocrinol Metab.

[59] MacArthur DG, Seto JT, Raftery JM, Quinlan KG, Huttley GA (2007). Loss of ACTN3 gene function alters mouse muscle metabolism and shows evidence of positive selection in humans.. Nat Genet.

[60] Quinlan KG, Seto JT, Turner N, Vandebrouck A, Floetenmeyer M (2010). Alpha-actinin-3 deficiency results in reduced glycogen phosphorylase activity and altered calcium handling in skeletal muscle.. Hum Mol Genet.

[61] Hakimi P, Yang J, Casadesus G, Massillon D, Tolentino-Silva F (2007). Overexpression of the cytosolic form of phosphoenolpyruvate carboxykinase (GTP) in skeletal muscle repatterns energy metabolism in the mouse.. J Biol Chem.

[62] Izumiya Y, Hopkins T, Morris C, Sato K, Zeng L (2008). Fast/Glycolytic muscle fiber growth reduces fat mass and improves metabolic parameters in obese mice.. Cell Metab.

